# Children then, adults now: long-term outcomes—performance at 15, 20, and 25 years of cochlear implant use

**DOI:** 10.3389/fresc.2023.1275808

**Published:** 2023-12-14

**Authors:** João Elói Moura, Jorge Humberto Martins, Marisa Alves, Graça Oliveira, Daniela Ramos, Helena Alves, Ricardo Caiado, António Teixeira, Luís Filipe Silva, Jorge Migueis

**Affiliations:** ^1^ Cochlear Implants Reference Center, Centro Hospitalar e Universitário de Coimbra, Coimbra, Portugal; ^2^ Department of Audiology, Center in Rehabilitation (CiR), School of Health, Polytechnic of Porto (ESS-P. Porto), Porto, Portugal; ^3^ Biomedical Informatics and Technologies (BIT), Institute of Electronics and Informatics Engineering of Aveiro (IEETA), Department of Electronics Telecommunications & Informatics, University of Aveiro, Aveiro, Portugal; ^4^ Serviço de Otorrinolaringologia, ENT Service, Centro Hospitalar e Universitário de Coimbra, Coimbra, Portugal

**Keywords:** cochlear implants, speech and language outcomes, congenital deafness, prelingual hearing loss, long-term outcomes

## Abstract

**Motivation:**

Severe to profound sensorineural hearing loss interferes with a child's development at the cognitive, linguistic, academic, and social levels. Since the beginning of the pediatric auditory rehabilitation program through cochlear implantation in the Ear, Nose, and Throat (ENT) Service of the Coimbra Hospital and University Center (CHUC), Portugal, its mentors defended the early diagnosis of hearing loss followed by timely intervention, and this was considered the starting point to optimize (re)habilitation through this method. Three decades or so later, recently we conducted this study to evaluate the performance of patients implanted in the initial phase of the cochlear implantation program.

**Objectives:**

The study aimed to evaluate the performance of individuals with severe to profound congenital hearing loss who underwent pediatric cochlear implantation and have used the cochlear implant for at least 25 years, to analyze the beneficial effect of early intervention in improving performance results.

**Methods:**

The study sample is composed of 31 individuals with severe to profound congenital hearing loss and no other comorbidities, divided into two groups (Group 1: age at implantation was under 3 years; Group 2: age at implantation was over 3 years). All 31 subjects were evaluated at 15, 20, and 25 years of cochlear implant (CI) use with a comprehensive set of tests. In addition, data were collected regarding the academic level of each participant. The results of both groups were compared to find out if there is an effect of age at implantation on auditory performance, and if there is an improvement in the performance with CI over time (15, 20, and 25 years of use).

**Results:**

The results show that there is a positive effect, with statistical significance, of early implantation on auditory performance, and telephone use. In both groups, there is an increase in performance over time, but it tends to stabilize after 20 years of CI use.

**Discussion and conclusion:**

The results obtained in this work support the importance of early intervention in patients with severe to profound hearing loss who are cochlear implant users and show that CI is an effective and reliable method in the treatment of these patients, contributing to their improved socio-educational integration, and that the benefits last over time.

## 1. Introduction

Since the 1990s, with the approval of cochlear implantation in children over 2 years of age by the Food and Drug Administration (FDA), the number of children who use cochlear implants (CIs) has been increasing. This fact, in parallel with the scenario of the expansion of the indication criteria for cochlear implantation in the pediatric population, the technological development in CI manufacturing, and assessment processes, has allowed the development of several research studies and clinical studies that seek to evaluate the results provided by cochlear implantation and explore the different variables that influence these results (1–6). There is also increasing scientific evidence of the influence of age at implantation on better speech, language and academic performances, influenced by brain plasticity, which has its critical acquisition period up to 4 years of age (7–9). The results obtained in the study published by Grandon et al. (10) show that (1) children with CIs have lower intelligibility, (2) early implantation is a predictor of good intelligibility, and (3) late implantation after two years of age does not prevent the children from eventually reaching good intelligibility (10).

In 2000, the FDA approved cochlear implantation in children aged 12 months and older (11) and, in 2020, the FDA changed the minimum age for bilateral cochlear implantation to 9 months of age, using specific cochlear implant equipment, in children with bilateral profound sensorineural deafness (12).

Follow-up studies of children after long periods of CI use become essential to inform professionals and families, both regarding therapy and expectations, as well as to better understand the factors involved in the process of developing the communicative, academic, and occupational skills of children who grew up using CIs (6, 13, 14). In their study from 2023, Gordon et al. confirm the importance of providing hearing through CIs early in development. The study also reveals the need for ongoing reporting of long-term effects of CIs in children given the remaining statistical uncertainties and the evolution of CI technology and candidacy (15). Waltzman et al. (16) presented a study with results that reveal significant gains in speech perception, use of oral language, and ability to function in a mainstream environment. In the same study, there was no decrease in performance over time and no significant incidence of device or electrode migration or extrusion, and device failure did not cause a deterioration in long-term outcome (16).

Some studies report that speech and language results remain stable in patients with more than 10 years of CI use (17, 18), or even up to 15 years (19). These authors also present data on the academic degree achieved by the patients who use CI, showing better results associated with early intervention. Geers et al. studied a group of teenagers who exhibited long-term benefits from cochlear implantation that extended into their high school years. Increases in performance were observed between elementary and high school for the students who attended mainstream classrooms and for students using primarily spoken language. Most of the teenagers were placed at an age-appropriate grade level in high school (20).

Beadle et al. (21) presented results suggesting that cochlear implantation provides long-term communication benefits to patients that do not plateau for some subjects even after reimplantation. The results further indicate that cochlear implant centers should create the structure and funding to provide long-term support, counseling, audiologic follow-up, rehabilitation, and device monitoring to every implanted child (21). In the study published by Angelika et al. (22), which presents data from implanted subjects with up to 17.75 years post implant (SD = 3.08; range 13–28), it was demonstrated that the majority of participants who underwent implantation at an early age achieved discrimination of speech sounds without lipreading. Educational, vocational, and occupational levels achieved by this cohort were significantly poorer compared with the German and worldwide population average. Children implanted today who are younger at implantation, and with whom more advanced up-to-date CIs are used, are expected to exhibit better auditory performance, and have enhanced educational and occupational opportunities (22).

In their study, Punch and Hyde (23) mention that the use of telephones, and in particular the mobile/cell phones, plays a key role in the social lives of many of these patients, being an integral part of their relationships with friends. Their findings indicate that many of the children and adolescents, even when they had been using cochlear implants since their first or second year of life, had difficulties using a telephone. Parents reported that their children would use the telephone with people they knew well, but struggled to converse, and lacked confidence, with people they were less familiar. For older adolescents, this could also be relevant for employment situations (23).

### 1.1. Pediatric cochlear implants program of the ENT Service at CHUC

Since the beginning of the pediatric cochlear implant program in the Otolaryngology Department of the (then) Centro Hospitalar de Coimbra, in 1992, the intervention through a multidisciplinary team, the early and timely process of cochlear implantation, and the intensive (re)habilitation were preponderant aspects for the program implementation. Regarding the team, it consisted of several otorhinolaryngologists with experience in ear surgery, special education teachers (later replaced by speech and language therapists), and audiologists, and there was a close collaboration with computer engineers, imaging doctors, neurodevelopment pediatricians, among other specialties. For early identification and intervention, and since the Service is also the Audiophonology Center of the Central Region of the country, a network was created for referring patients by general practitioners, schools, and other ENT Departments, which allowed patients to arrive at CHUC at earlier ages. The implementation of this rehabilitation method motivated the team to create an intervention program that involved intensive training with the child staying in the department for an average period of 3 months, during which the programming of the speech processor was carefully conducted, and intensive sessions were carried out to maximize the auditory, language, and speech development. After those average first 3 months of intervention, a first assessment was carried out and the patient returned to his area of residence, where he/she would have speech therapy and special education. Then, the patient would return to the center for new assessments at 6, 9, 12, 18, and 24 months after the activation of the speech processor. After 24 months, and depending on the need for new patient assessments, they would return to the center once a year, at 3, 4, 5, …, 15, 20, and 25 years after the activation of the speech processor. Whenever it was necessary for patients to come in other moments or stay for longer periods of time for intensive sessions, the patient's situation was studied individually so that the best response to the situation could be arranged. This approach to the post CI (re)habilitation process has remained similar over time, although keeping up with the advances in technology and intervention approaches.

## 2. Methods

The study is an exploratory retrospective, in which the performance of the patients in the sample was compared at 15, 20, and 25 years of cochlear implant use, with the patients having been divided into two groups: Group 1 with implant age equal to or less than 36 months, and Group 2 aged over 36 months when implanted.

The following assessment instruments were used: Monosyllables, Numbers, and Sentences Tests (24), Sentences on the telephone test, Common words test, Common words on the telephone test, Minimal Pair Discrimination test (25), and Consonant test (26). In addition, data were collected regarding the academic level of each participant.

The patients were asked to listen and repeat each of the tests’ stimuli. The tests were presented in a soundproof room with the patient sitting one meter away. The number of correct answers was retained, and the percentage of correct answers was obtained dividing it by the number of stimuli integrating the test.

Through the assessments, the following questions were examined:

Q1: Is there a positive effect of early cochlear implantation on the auditory performance of children (now adults) who use cochlear implants?

Q2: Is there performance improvement even after 10 years of cochlear implant use?

Q3: Is there an effect of early implantation on telephone use performance?

Q4: If there is a positive effect of early cochlear implantation on auditory performance, is that effect similar for all assessed skills?

### 2.1. Inclusion and exclusion criteria

The following inclusion criteria were adopted for the study:

− Patients with severe to profound congenital hearing loss without benefit from the use of hearing aids, implanted between 1992 and 1997.
− Only patients whose assessments were carried out at the three defined moments (15, 20, and 25 years of CI use) were included in the sample.

The exclusion criteria were the following:

− Patients who did not attend one or more of the assessments at the three defined moments.
− Patients whose implanted device was replaced.
− Patients with acquired hearing loss.
− Patients who are non-users.

### 2.2. Sample characterization

All the patients with severe to profound hearing loss who received a cochlear implant when they were children, between 1992 and 1997, at the Cochlear Implants Reference Center of the CHUC (*n* = 51) were identified. Of the 51 identified patients, two had an implanted device with malfunctions that was replaced by another more recent model of the same brand, and hence they were excluded from the sample. Twelve patients had severe to profound acquired sensorineural hearing loss, so they did not meet the inclusion criteria and were also not part of the sample. Six other patients were not included in the study for the following reasons: three were not using the implant (two by their own choice and one had it explanted for medical reasons) and the remaining three had not had all three evaluations performed. Therefore, of the initial 51 identified patients, 31 met the inclusion criteria and were selected to be included in the sample. All selected participants were Caucasian and native speakers of Portuguese, originating from various regions of Portugal, including the North, Center, Lisbon, Algarve, and Madeira. These 31 patients were divided into two groups: Group 1 with age at cochlear implantation equal to or less than 36 months (*N* = 17,), and Group 2 with age at cochlear implantation greater than 36 months (*N* = 14). All patients included in the sample use the same stimulation strategy (SPEAK) and stimulation mode (BP + 1).

Table 1 presents the data characterizing each evaluated group according to the variables “gender,” “implant side,” “mean age at the time of cochlear implantation,”, and “academic level.”

**Table 1:** Characterization of the evaluated groups regarding gender, implant side, mean age at the time of cochlear implantation, and academic level.

	Gender		Implant side		Mean age at CI (months)	Academic level			
	Male	Female	Right	Left		High school	University student	University degree	Master's degree
Group 1 ≤ 3 years (*N* = 17)	6	11	17	0	34 ± 2.20	8	4	3	2
Group 2 > 3 years (*N* = 14)	6	8	12	2	45 ± 4.97	9	3	1	1

Table 2 presents the data regarding the CI model and speech processor used.

**Table 2:** Sample characterization regarding the CI model and speech processor used.

	Cochlear implant model			Speech processor	
	Group 1	Group 2		Group 1	Group 2
Cochlear CI22	4	5	CP810	2	2
CI22M	11	6	CP910-22	9	6
CI24M	2	1	CP1000-22	6	5
CI24ST		1			

### 2.3. Statistical analyses

The obtained sample data were subjected to a descriptive statistical analysis (mean and median values, standard deviation, and their variation with several factors) and comparative statistical analyses, performed in R.

Owing to the type of data (percentages derived from counts of correct answers), the statistical analyses followed (27), also adopting Generalized Linear Models (GLM) and using the function glm() in R with a Poisson distribution. The significance level adopted was 0.01.

## 3. Results

In this section, the results will be organized according to the four research questions presented in Section 2, starting with the more general question regarding the overall effect of early implantation.

### 3.1. There is a positive effect of early cochlear implantation on the auditory performance of children (now adults) who use cochlear implants

**Figure 1:**
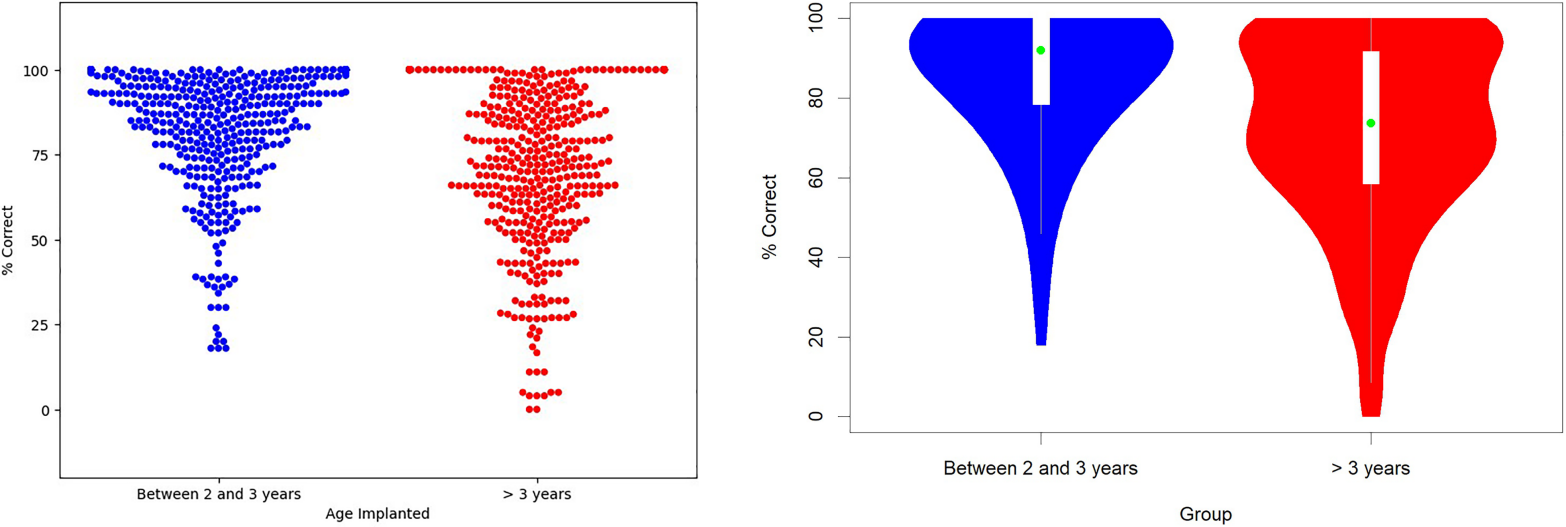
Distribution of auditory tests’ results for subjects implanted before and after 3 years of age. Left: the distribution of all obtained test results; right: the estimates of the distributions in the form of a violin plot.

To answer the first and main research question: if there is a positive effect of early implantation on the auditory performance of implanted children (now adults), the distribution of auditory tests’ results for subjects implanted before and after 3 years were compared. The results are presented in Figure 1, which shows both the distribution of real results (at the left) and an estimate of distribution through a violin plot (at the right). To provide more information regarding the results, we opted for the presentation of the detailed distribution of the values instead of the more common boxplot. To complement the information presented in the graphs and allow additional quantitative comparisons, several descriptive statistics are presented for the two groups in Table 3. To complement the information on central tendency of the results given by the mean, Table 3 also includes the median, more robust to outliers.

**Table 3:** Descriptive statistics for the auditory tests’ results for subjects implanted before and after 3 years of age.

	Mean	Median	First quantile	Standard deviation
Group 1 (implanted before 3 years of age)	85.8	92.0	78.4	17.5
Group 2 (implanted after 3 years of age)	71.4	73.7	58.4	23.7
Group 1 − Group 2	14.4	18.3	20.0	

For each group, two measures of central tendency (mean and median), a measure of dispersion (standard deviation), and a measure related to the lower values attained (1st quantile) are presented. In addition, in the last row, the differences between the two groups are presented.

By analyzing Figure 1 and Table 3, we can verify that the overall results obtained by patients implanted before 3 years of age show better performance in the tests than the individuals with age at cochlear implantation more than 3 years. Despite the results’ dispersion for both groups, it is clear in both Figure 1 graphs that the results are more concentrated in higher values for the patients with earlier cochlear implantation. There is a higher concentration of values on the left side (Group 1) above 75% compared with the right side (Group 2), which has the most dispersed values, with a considerable number of results around 50%. Also, the left side (Group 1) has no values close to zero.

Differences between the two groups are also clear in the descriptive statistics presented in Table 3, particularly the medians, which differ by almost 20%.

The significance of the differences between the two groups was assessed by adopting a GLM Poisson model with one factor. Results confirmed the differences as significant, with *p* < 0.001.

All these results indicate a clear advantage of implantation before 3 years of age, but there is the possibility of this being due to confounding factor(s). One factor that could be affecting the results is academic training. The analyses considering education as a factor are presented next.

No statistical tests were conducted with academic level as a factor due to the limited number of participants in Group 2 holding university and Master's degrees (only 2).

#### 3.1.1. Academic level effect

**Figure 2:**
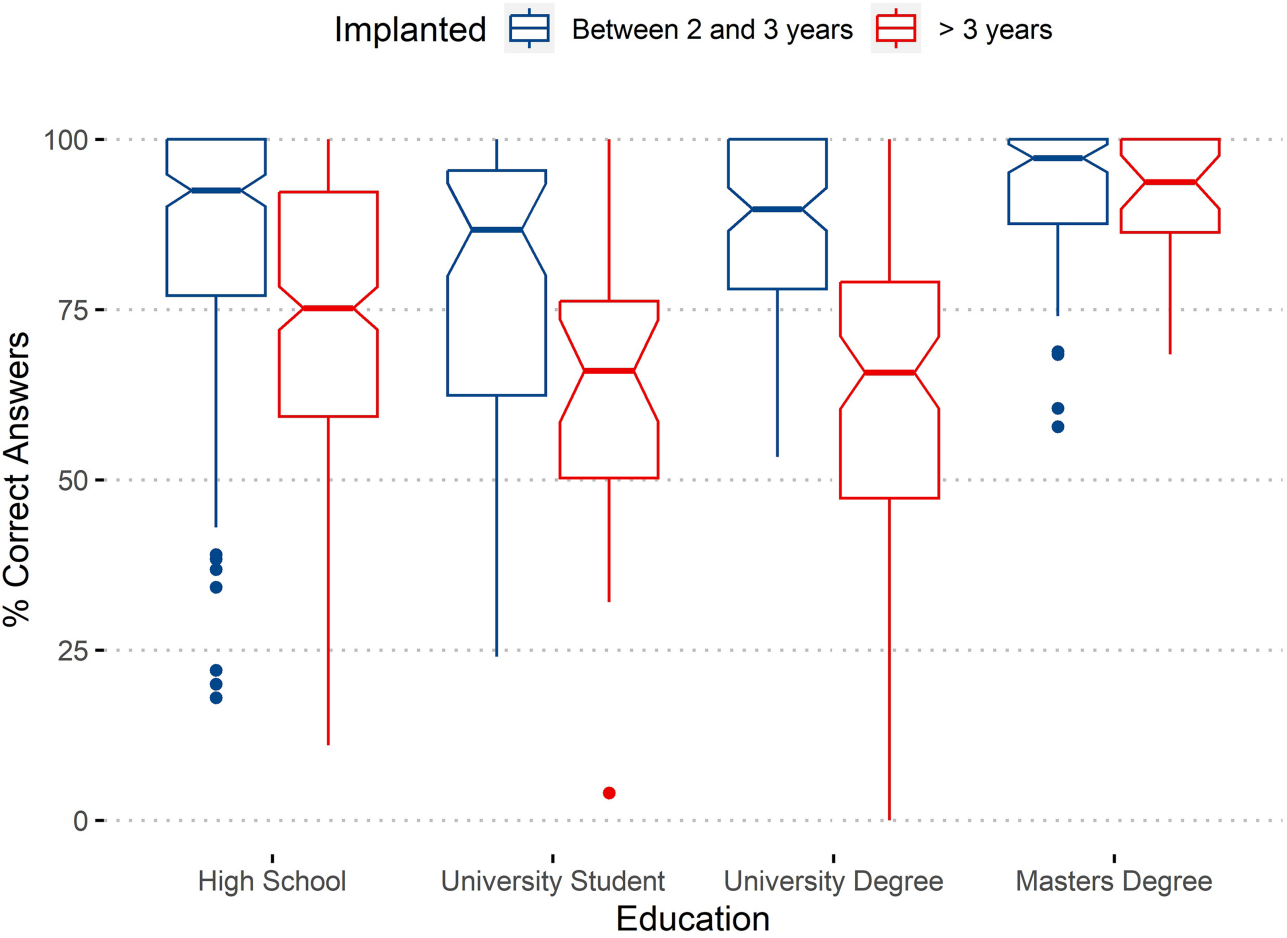
Effect of age of implantation and education (academic level) on percentage of correct answers.

In Figure 2, we can observe the effect of age at cochlear implantation on the tests’ performance separated by academic level. The figure summarizes the results using boxplots showing not only the quartiles but also information on 95% confidence intervals (as notches).

By analyzing the results presented in Figure 2, we can verify that children implanted earlier achieve noticeable differences in results, except for those who finish a Master's degree (which are only a few). The non-superposition of notches confirms the differences as significant, at a 95% confidence level.

Worth mentioning is the fact that, although the group of children with a higher age at implantation show results that are worse comparatively, some of them had succeeded in earning college degrees, including a Master's degree.

### 3.2. Performance improves even after 10 years of cochlear implant use

A second relevant question is whether performance improves over time and if the effect of age at implantation is maintained.

**Figure 3:**
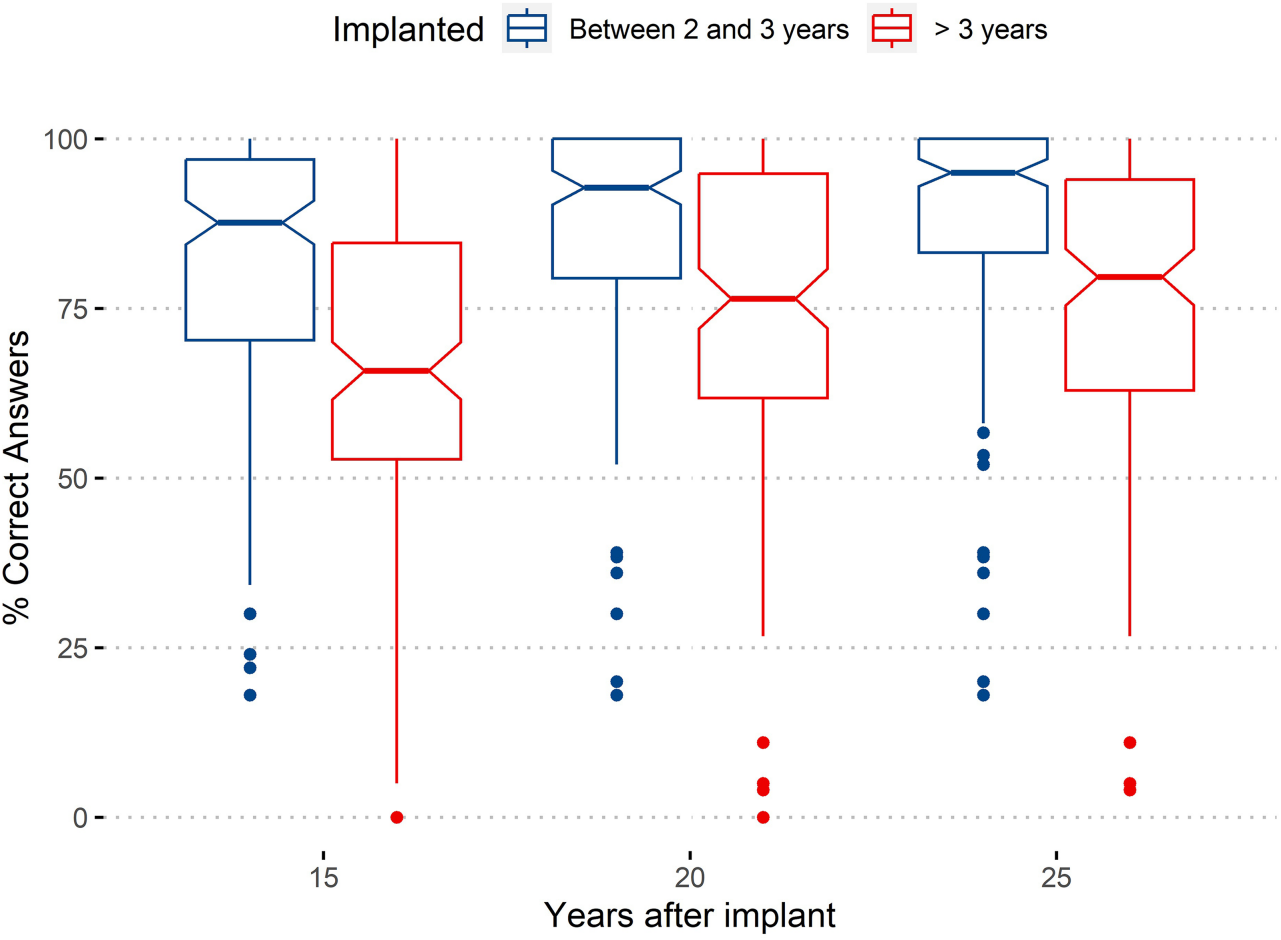
Comparison of the percentage of correct answers obtained for both groups in the three evaluation stages (15, 20, and 25 years after implantation).

Figure 3 compares the overall results of the tests obtained for both groups at all three assessment stages (15, 20, and 25 years after implantation). Complementing the figure, Table 4 provides statistical measures (mean and median) for the two groups at the three evaluation stages as well as the differences between the two groups/ages of implantation for the three evaluations.

**Table 4:** Descriptive statistics (mean and median) for the percentage of correct answers considering both age of implantation (rows) and number of years after implantation (columns).

	Mean			Median		
	15 years	20 years	25 years	15 years	20 years	25 years
Group 1 (implanted before 3 years of age)	81.6	87.1	88.8	87.7	92.8	95.0
Group 2 (implanted after 3 years of age)	65.8	73.5	75.2	65.8	76.4	79.6
Group 1 − Group 2	15.8	13.6	13.6	21.9	16.4	15.4

In addition, the differences between the two groups regarding age of implantation are presented in the last row.

By observing Figure 3, we can see that the overall performance in the tests improves in both groups, although less from 20 to 25 years. We can verify that the performance at any of the assessment moments is always better in the group of CI users implanted earlier. The non-superposition of notches confirms as significant the differences between the two groups at the three evaluations (15, 20, and 25 years), at a 95% confidence level.

To assess the significance of the effects of this new factor (years after implant), a new, one-factor, GLM Poisson model was applied. The results improve significantly with years after implantation. A *post-hoc test* for the factor “years after implant” revealed significantly different results for the three levels. The results after 20 and 25 years of implant are significantly better than those obtained after 15 years, with a *p*-value lower than 0.001; the positive difference from 20 to 25 years is also significant but has a lower *p*-value (0.0393).

The one-factor analysis was complemented by a two-factor analysis, considering years after implantation and age of implantation. The results confirmed the differences for the twofactors as significant, with *p* < 0.001, and the difference between the two ages of implantation was also significant considering “years after implant” as an additional factor. Furthermore, the results also revealed a significant interaction effect of implantation age and years after implantation (*p* < 0.01), a sign that the difference between age of implantation is affected by years after implant, with the difference decreasing slightly with the increase of years.

### 3.3. Earlier age at cochlear implantation has a positive effect on the performance of tests through the telephone

Conversations on the phone, watching TV, and enjoying listening to music are some of the most complicated tasks for patients with cochlear implants. In CI rehabilitation sessions, conversations on the telephone are one of the most difficult and later achieved objectives as mentioned in the work of Punch and Hyde (23).

**Figure 4:**
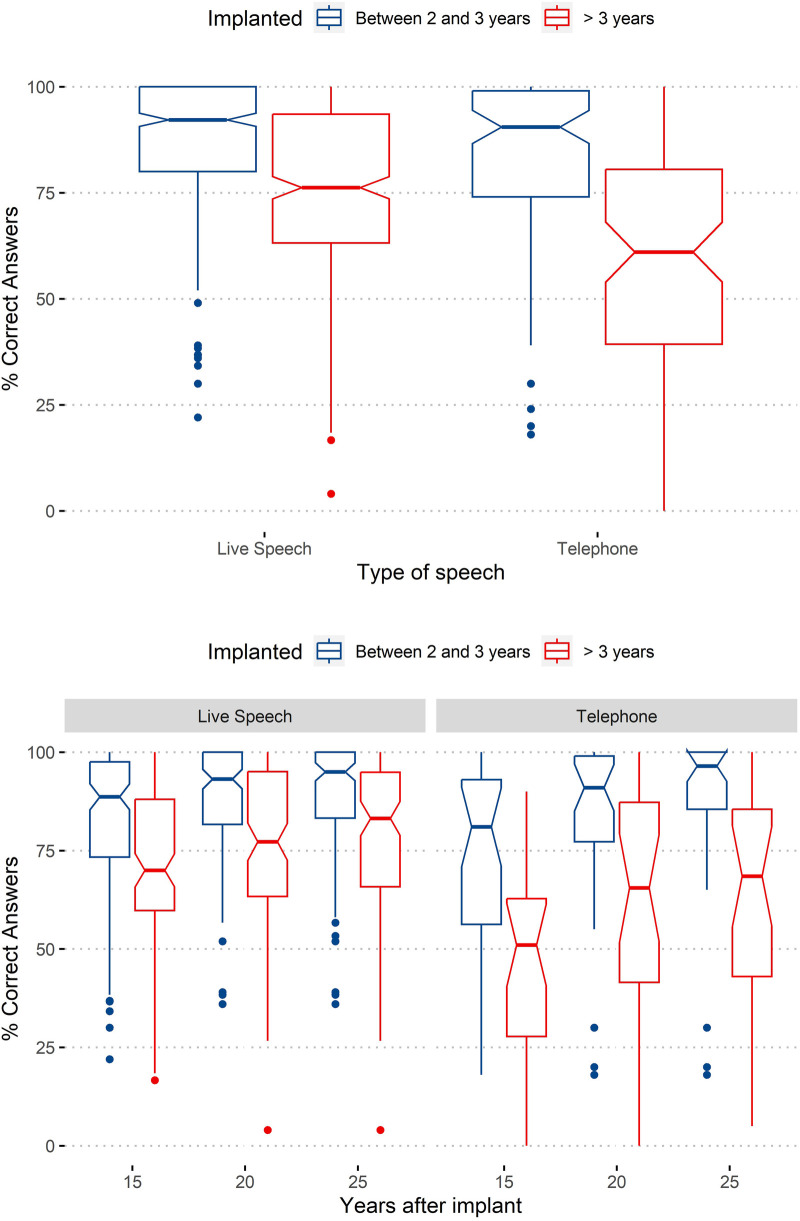
Effect of type of speech (live and through the telephone) on the percentage of correct answers. Top: boxplots considering two factors (age of implantation group and type of speech); bottom: boxplots considering an additional third factor (years after implant).

Aiming to know more about the effect of the age at implantation on the performance in tasks involving the telephone, Figure 4 presents the results obtained in the two assessment conditions (voice tests through the telephone and live voice tests) for both groups.

By observing Figure 4, we can verify that the results obtained for the group of users implanted before they were 3 years old are similar for voice tests through the telephone and live voice tests; with voice through the telephone, we have clearer differences between groups. The figure also shows a higher dispersion of the results for voice tests through the telephone, especially for tests performed with the group of users implanted after the age of 3 years. In addition, the gap between both groups tends to reduce with years after implantation, with the gap being much higher for telephone voice tests.

In line with the procedure in the previous subsection, to assess the significance of the effects of this new factor (Type of Speech), a GLM Poisson model was applied. Results confirmed as significant the effect of type of speech, with *p* < 0.001.

The one-factor analysis was complemented by a two-factor analysis, also considering age of implantation. Results confirmed the differences for the two factors as significant, with *p* < 0.001, and the difference between the two ages of implantation was also significant when considering “type of speech” as an additional factor. The interaction of type of speech with age of implantation was also significant (*p* < 0.001).

### 3.4. Early cochlear implantation leads to better results in most assessed abilities

**Figure 5:**
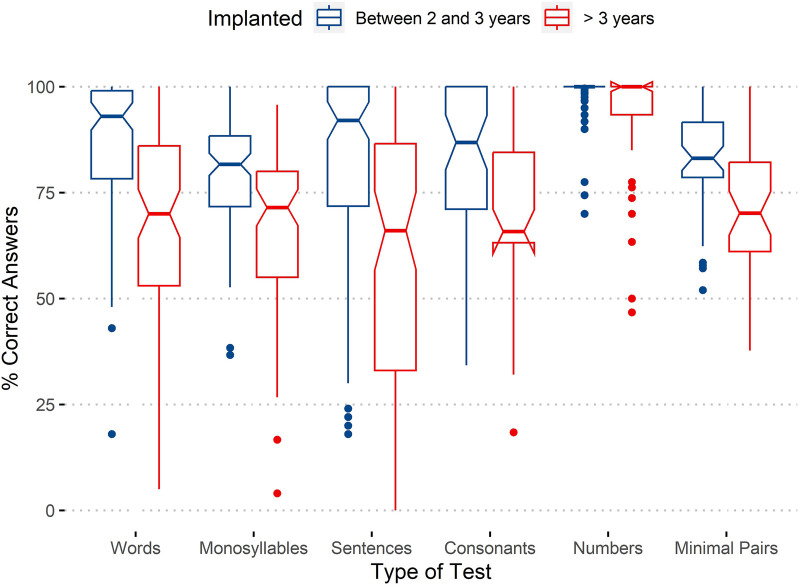
Percentage of correct answers by age of implantation and type of test.

As assessment tests target different abilities, it is also relevant to investigate the effect of age at implantation with Type of Test as an additional factor. Continuing to use boxplots, the results as function of assessment test and age of implantation are presented in Figure 5.

Regarding means, the beneficial effect of implantation before 3 years of age is clear for most tests, except Numbers, in which both groups reach 100%. This scenario remains when considering the 95% confidence interval, showing all test results, except Numbers, as significantly better for the group implanted earlier.

The tests with the worst results are the Monosyllables test and Minimal Pair Discrimination test. These tests present means below 75% for the group implanted after 3 years of age.

The results for the Sentences test present one of the greatest differences between both the groups (alongside Consonants test) together with a greater dispersion of values for the group implanted later.

From the combination of the aforementioned results, the beneficial effect is comprehensive and is not limited to a subset of the evaluated abilities.

As in previous sections, the effect of the new factor (Type of Test) was confirmed as significant (*p* < 0.001) by a one-factor GLM Poisson model. A *post-hoc test* revealed the differences among all pairs of tests were significant (*p* < 0.001), except for the pair “Numbers – Consonants” (*p* = 0.906).

### 3.5. Joint analysis of the factors

To complement the one- and two-factor statistical analysis reported in the previous sections, a four-factor GLM Poisson model was applied. The factors considered were age at implantation, years after implant, type of speech, and type of test. The results confirmed the effects of all factors were significant, corroborating the univariate analysis.

**Figure 6:**
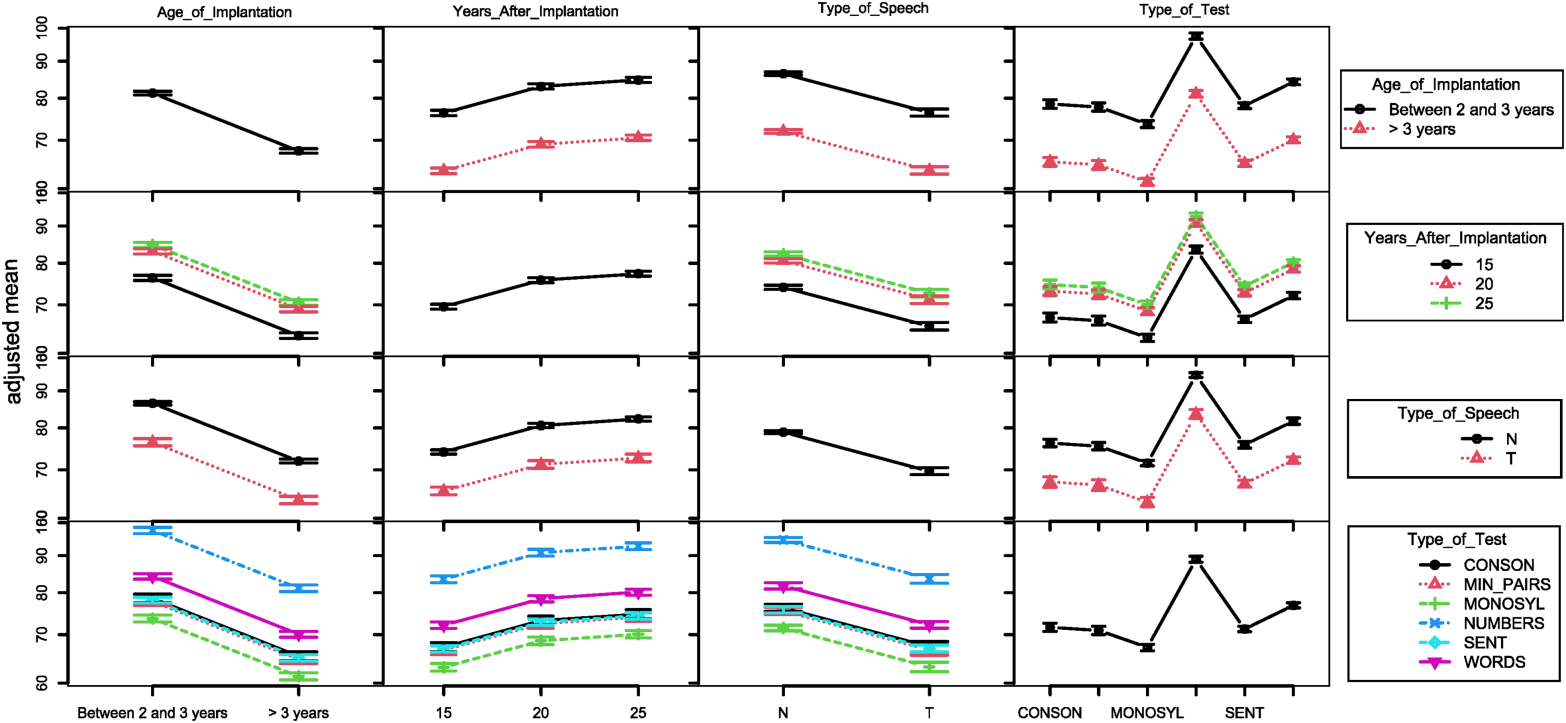
Interaction plot for the several factors with potential influence in the test results. The following are presented, from left to right (and top to bottom): age of implantation (2 groups), time of evaluation years after implantation, type of speech (T for telephonic speech, N otherwise), and type of test.

The effects of the several factors in the tests’ results are summarized in the interaction plot of Figure 6 created using the function interactionMeans() of phia R library^1^.

The plots in the diagonal point to (1) differences between groups implanted before and after 3 years of age, with better results for the former; (2) some variation regarding academic accomplishments; (3) increase of performance with years of use; (4) better performance with live speech tests; (5) variation of the results with the type of test.

The first row, combining age at implantation with the other factors, shows that results for the earlier age at implantation (at right) are higher for all situations.

## 4. Discussion

Nowadays, the auditory rehabilitation of severe to profound congenital sensorineural hearing loss through cochlear implantation is a consolidated technique accepted worldwide, which allows the acquisition and development of oral language close to normality (6, 28). The obtained results, revealing better performance in the evaluation tests for the group of children implanted earlier, are in agreement with the results found in the literature (8, 9, 20), providing additional support for the benefits of early implantation and initiation of rehabilitation (6, 29).

Another general trend in the obtained results is the positive influence of time of use on performance. The obtained results show that both groups present an improvement in performance over time at least up to 20 years after cochlear implantation, with an improvement in performance from 15 to 20 years of use and a stabilization of performance in the period between 20 and 25 years. These results are not in line with the results published by Geers and Sedey (19), which indicate the existence of a stabilization of results after 10 years of use. This difference may be related to the improvement of speech processors and motivates us to continue investing in intervention in these patients until later ages, especially in periods when speech processors are upgraded, to maximize performance with the newer technology.

With the growing needs for social and professional interaction through the telephones or mobile phones, the results of the tests conducted over the telephone are very relevant. They follow the general trend, also showing a higher performance in the group of children implanted earlier and an improvement from 15 to 20 years of CI use. These results present a better performance than the results presented by Punch and Hyde (23). However, we cannot forget that, since that date, there has been a significant improvement in telecommunications equipment, speech processors, and the interaction between them, which could have positively influenced our results.

The higher performance of the group with earlier age at implantation is not restricted to a limited set of assessed abilities, since the results for all the six tests used confirm the higher performance of this group.

Regarding the performance in the tests according to the academic level, we can see that the group of individuals implanted at an earlier age also presents a better average performance in the used tests.

### 4.1. Limitations of the study

Despite the great wealth of data that support this work, covering 25 years and more than 1,400 implants, there are some biases in the study, the main ones being: (1) implant side, overwhelmingly on the right side; (2) use of only one stimulation strategy (SPEAK) and stimulation mode (BP + 1), although several models of implants were used; (3) unilateral implant. However, we consider that they do not compromise the study and are perfectly justifiable: implantation in the right ear is the best practice for unilateral cochlear implantation, when both ears show similar characteristics; the selected stimulation strategy and mode constituted the best solution available at the time of these implantations; bilateral implants only appeared later (the first implant of this type in the center where the study took place was carried out in 2007, 15 years after the first pediatric implant in our sample).

As there have been updates to the processors over time, to lessen the negative effect that these patients’ access to different acoustic characteristics could have, the assessment of the patients in our sample was conducted at least 1 year after the processor upgrade. However, it was not possible to control other factors, such as the socio-economic environment of the participants.

A final limitation of the study is the adoption of a single follow-up, rehabilitation, and evaluation method (Pediatric cochlear implants program of the ENT Service of CHUC). In this way, it is not possible to generalize results such as the improvement 20 years after implantation without studies by other teams adopting alternative approaches. Eventually, this improvement may be at least partially related to the method and not just to the evolution of processors mentioned previously.

## 5. Conclusion

The results obtained support the hypothesis that the cochlear implant is an effective method in the treatment of severe to profound hearing loss and that the results obtained are positively influenced by early intervention. The results also show that there may be performance improvement after long years of use and that the follow-up and support of these patients is reflected in their success.

## Data Availability

The original contributions presented in the study are included in the article/Supplementary Material, further inquiries can be directed to the corresponding authors.

## Footnotes

^1^https://www.rdocumentation.org/packages/phia/versions/0.2-1/topics/interactionMeans.
